# 484. Identification of Early Features to Differentiate Hospitalized Children Admitted for Suspected MIS-C from Alternative Diagnoses

**DOI:** 10.1093/ofid/ofab466.683

**Published:** 2021-12-04

**Authors:** Matthew T Clark, Danielle A Rankin, Anna E Patrick, Alisa Gotte, Alison Herndon, William McEachern, Andrew Smith, Mary Ann Thompson, M D Moore, Joseph R Starnes, Jessica Anderson, Melanie Whitmore, Kathy Jabs, Rebecca Kidd, Heather L McDaniel, Ryan Wolf, Daniel E Clark, Giovanni Davogustto, Edward Hardison, Quinn Wells, David Parra, Natasha B Halasa, Natasha B Halasa, James A Connelly, Sophie E Katz

**Affiliations:** 1 Vanderbilt University Medical Center, Nashville, TN; 2 Vanderbilt University Medical Center; Division of Pediatric Infectious Diseases, Nashville, TN; 3 Vanderbilt, Nashville, TN; 4 VUMC, Nashville, TN; 5 Johns Hopkins All Children’s Hospital, St. Petersburg, Florida; 6 Monroe Carell Jr. Children’s Hospital at Vanderbilt, Nashville, TN

## Abstract

**Background:**

Multi-system inflammatory syndrome in children (MIS-C) is a rare consequence of severe acute respiratory syndrome coronavirus 2 (SARS-CoV-2). MIS-C shares features with common infectious and inflammatory syndromes and differentiation early in the course is difficult. Identification of early features specific to MIS-C may lead to faster diagnosis and treatment. We aimed to determine clinical, laboratory, and cardiac features distinguishing MIS-C patients within the first 24 hours of admission to the hospital from those who present with similar features but ultimately diagnosed with an alternative etiology.

**Methods:**

We performed retrospective chart reviews of children (0-20 years) who were admitted to Vanderbilt Children’s Hospital and evaluated under our institutional MIS-C algorithm between June 10, 2020-April 8, 2021. Subjects were identified by review of infectious disease (ID) consults during the study period as all children with possible MIS-C require an ID consult per our institutional algorithm. Clinical, lab, and cardiac characteristics were compared between children with and without MIS-C. The diagnosis of MIS-C was determined by the treating team and available consultants. *P*-values were calculated using two-sample t-tests allowing unequal variances for continuous and Pearson’s chi-squared test for categorical variables, alpha set at < 0.05.

**Results:**

There were 128 children admitted with concern for MIS-C. Of these, 45 (35.2%) were diagnosed with MIS-C and 83 (64.8%) were not. Patients with MIS-C had significantly higher rates of SARS-CoV-2 exposure, hypotension, conjunctival injection, abdominal pain, and abnormal cardiac exam (Table 1). Laboratory evaluation showed that patients with MIS-C had lower platelet count, lymphocyte count and sodium level, with higher c-reactive protein, fibrinogen, B-type natriuretic peptide, and neutrophil percentage (Table 2). Patients with MIS-C also had lower ejection fraction and were more likely to have abnormal electrocardiogram.

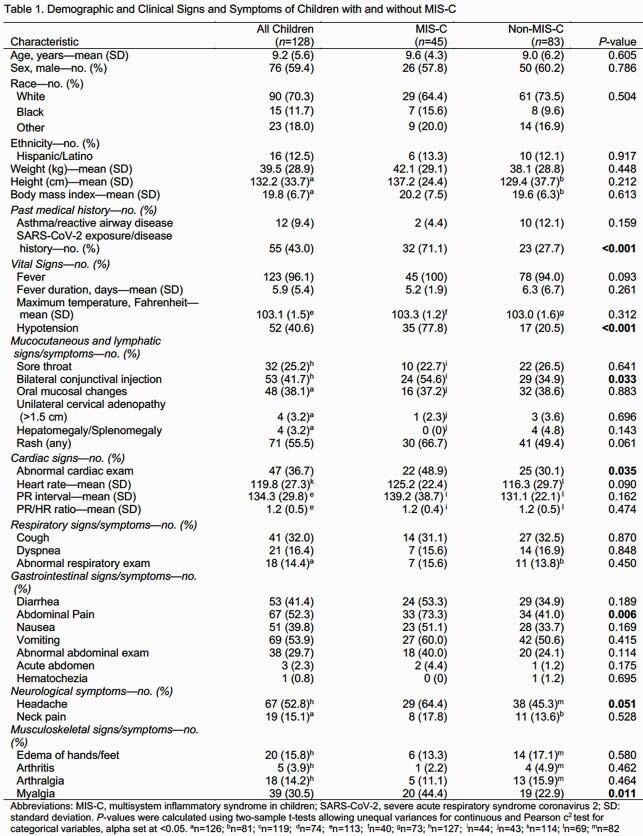

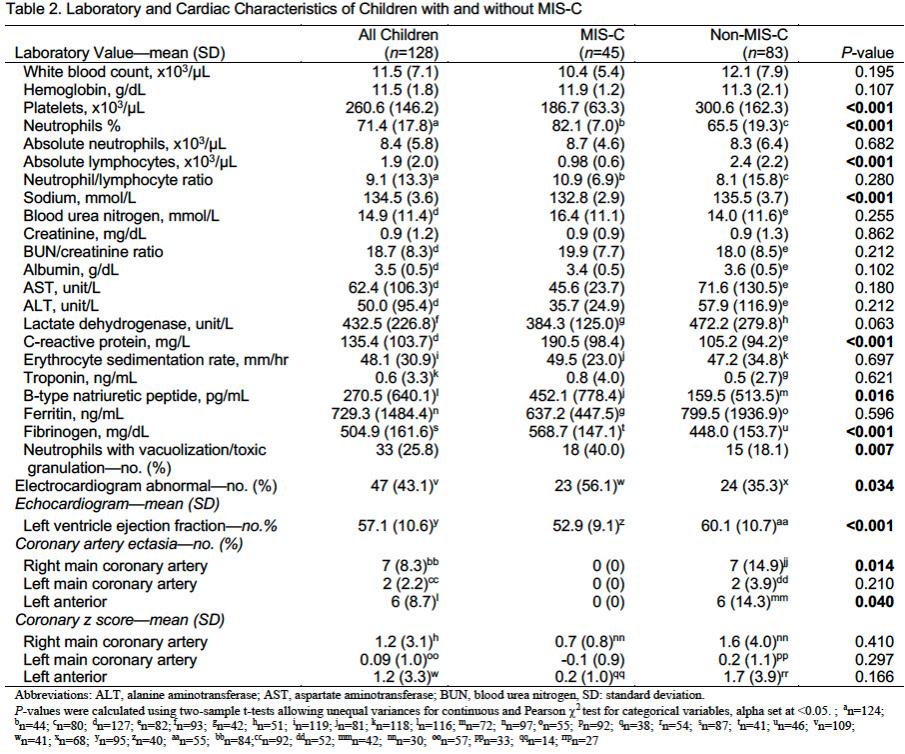

**Conclusion:**

We identified early features that differed between patients with MIS-C from those without. Development of a diagnostic prediction model based on these early distinguishing features is currently in progress.

**Disclosures:**

**Natasha B. Halasa, MD, MPH**, **Genentech** (Other Financial or Material Support, I receive an honorarium for lectures - it’s a education grant, supported by genetech)**Quidel** (Grant/Research Support, Other Financial or Material Support, Donation of supplies/kits)**Sanofi** (Grant/Research Support, Other Financial or Material Support, HAI/NAI testing) **Natasha B. Halasa, MD, MPH**, Genentech (Individual(s) Involved: Self): I receive an honorarium for lectures - it’s a education grant, supported by genetech, Other Financial or Material Support, Other Financial or Material Support; Sanofi (Individual(s) Involved: Self): Grant/Research Support, Research Grant or Support **James A. Connelly, MD**, **Horizon Therapeutics** (Advisor or Review Panel member)**X4 Pharmaceuticals** (Advisor or Review Panel member)

